# Cranioplasty with autologous cryopreserved bone after decompressive craniectomy. Complications and risk factors for developing surgical site infection

**DOI:** 10.1007/s00701-013-1992-6

**Published:** 2014-02-04

**Authors:** J. Sundseth, A. Sundseth, J. Berg-Johnsen, W. Sorteberg, K.-F. Lindegaard

**Affiliations:** 1 Department of Neurosurgery, Oslo University Hospital Rikshospitalet, Postboks 4950, Nydalen, 0424 Oslo, Norway; 2 Institute of Clinical Medicine, University of Oslo, Oslo, Norway; 3 Department of Neurology, Medical Division, Akershus University Hospital, Lørenskog, Norway

**Keywords:** Cranioplasty, Craniectomy, Autologous, Cryopreserved, Surgical site infection, Complications

## Abstract

**Background:**

Renewed interest has developed in decompressive craniectomy, and improved survival is shown when this treatment is used after malignant middle cerebral artery infarction. The aim of this study was to investigate the frequency and possible risk factors for developing surgical site infection (SSI) after delayed cranioplasty using autologous, cryopreserved bone.

**Methods:**

This retrospective study included 74 consecutive patients treated with decompressive craniectomy during the time period May 1998 to October 2010 for various non-traumatic conditions causing increased intracranial pressure due to brain swelling. Complications were registered and patient data was analyzed in a search for predictive factors.

**Results:**

Fifty out of the 74 patients (67.6 %) survived and underwent delayed cranioplasty. Of these, 47 were eligible for analysis. Six patients (12.8 %) developed SSI following the replacement of autologous cryopreserved bone, whereas bone resorption occurred in two patients (4.3 %). No factors predicted a statistically significant rate of SSI, however, prolonged procedural time and cardiovascular comorbidity tended to increase the risk of SSI.

**Conclusions:**

SSI and bone flap resorption are the most frequent complications associated with the reimplantation of autologous cryopreserved bone after decompressive craniectomy. Prolonged procedural time and cardiovascular comorbidity tend to increase the risk of SSI.

## Introduction

Decompressive craniectomy is a lifesaving surgical procedure in acute brain swelling [12, 49]. The technique was first described by Harvey Cushing in 1905 [10]. Common causes of acute brain swelling are malignant middle cerebral artery (MCA) infarction, aneurismal subarachnoid hemorrhage (aSAH), intracerebral hemorrhage (ICH), traumatic brain injury (TBI), acute subdural hematoma (ASDH), and reactive edema after intracranial surgery. The procedure was first introduced for MCA infarction in 1956 [44].

For those who survive, the cranial defect after decompressive craniectomy is usually reconstructed at a later point of time. Cranioplasty is performed for the following reasons: protection of the brain, restoration of the brain's hydrodynamic conditions [14, 29, 51, 52], and cosmetics. Cranioplasty can be performed by using autologous bone or patient-specific prostheses made of various alloplastic materials [6, 9, 23, 31]. Autologous bone is often preferred to allograft due to its perfect match to the bony defect and low cost.

Surgical site infection (SSI), defined as infection resulting in surgical removal of the implanted bone, is a feared complication in delayed cranioplasty. This retrospective study was conducted to identify complications after cranioplasty with cryopreserved autologous bone and to search for possible risk factors associated with the development of SSI.

## Material and methods

### Patients

The study includes all patients from over a period of 12.5 years (May 1998 to October 2010) that underwent (1) decompressive craniectomy for acute brain swelling, (2) cryopreservation of the removed bone flap, and (3) reimplantation of the bone flap at the Neurosurgical Department, Oslo University Hospital Rikshospitalet, Oslo, Norway. Rikshospitalet is a tertiary-level university hospital that does not manage trauma patients.

Patients who had their reimplanted cryopreserved bone flaps removed due to SSI or bone flap resorption later underwent a secondary cranioplasty using a prefabricated, patient-specific prosthesis made of porous, biocompatible polyethylene (Medpore biomaterial customized implant. Porex Surgical, Inc. 15 Dart Road, Newnan, GA 30265-1017 USA).

### Methods

#### Surgical procedures

##### Decompressive craniectomy

In the decompressive craniectomy procedure, a standard frontotemporoparietal hemicraniectomy was performed, whereupon the dura mater was opened in a stellate fashion. A dura substitute (Neuro-Patch® Aesculap AG, Am Aesculap-Platz, 78532 Tuttlingen) was then placed over the cerebral cortex, and the opened dura was repositioned over the substitute. In the past few years, a second dura substitute was positioned between the dura and the temporal muscle in order to ease surgical dissection of the temporal muscle during cranioplasty. The subcutis and cutis were closed separately. Connective tissue and blood were removed from the bone flap. The flap was immediately thereafter washed in sterile H_2_O_2_ and NaCl 9 mg/ml dried with sterile gauze, packed in a two-layer sterile instrument pouch (3 M Health Care 9097 St. Paul, MN 55144–1000 USA), and placed in an ultra-low freezer (Forma Scientific Inc. SA mod. 925, 401 Mill Creek Rd, Marietta, OH 45750) at - 86 °C.

##### Primary cranioplasty

At primary cranioplasty, the bone flap was retrieved from the freezer when the patient arrived in the operating theater and then was thawed at room temperature. The sterile instrument pouch was opened and the bone placed in sterile Gentamycin-/NaCl solution. The cutis and subcutis were cut open corresponding to previous incisions and dissected from the cranium and the temporal muscle. The temporal muscle was carefully dissected from the dura, avoiding any CSF leak. The edges of the scull at the previous craniectomy site were trimmed using a drill and a diamond burr so that the cranium and the bone flap matched exactly. The bone was then put into place and fixed to the scull using a craniofix (B Braun Aesculap Tuttlingen Germany) and/or the Lorenz plating system (Biomet microfixation 1520 Tradeport Drive Jacksonville FL 32218–2480). The temporal muscle was reattached to the temporal bone with sutures against small, separate burr holes when it was straightforward in order to dissect the muscle. In cases where the muscle was adherent and difficult to release from the dura, the bone flap was replaced over the muscle. The selected method was dependent on the individual surgeon’s choice. Eight different surgeons with varying levels of experience have performed the primary cranioplasty procedures on the patients in this study.

##### Secondary cranioplasty

During secondary cranioplasty, the surgical procedure until adequate exposure of the cranial defect was performed as described during the primary cranioplasty. A prefabricated patient-specific prosthesis was trimmed and fitted to match the cranial vault exactly and fixed to the scull using the Lorenz plating system, as described previously by the authors [47].

#### Data obtained from the patients’ medical journals

The following data were obtained retrospectively from the patients’ medical journals: age, sex, smoking habits, previous medical history, etiology of the brain swelling leading to decompressive craniectomy, side of craniectomy, prophylactic antibiotic treatment, duration of the primary cranioplasty surgical procedure (minutes), subcutaneous drainage after cranioplasty, in-hospital infection, time from decompressive craniectomy to cranioplasty (days), and follow-up (months).

The complications recorded after cranioplasty were SSI, post-operative hematoma, and bone resorption resulting in repeat surgery.

### Ethical considerations

This study is part of a retrospective follow-up trial on patients with MCA infarction, approved by the Regional Committee for Medical and Health Research Ethics, and the data protection official for research. The present analysis was separately evaluated by the Regional Committee for Medical and Health Research Ethics as a quality assurance of existing treatment, and an additional special committee approval was therefore not needed. The study was also approved by the data protection official for research.

### Statistical analysis

Categorical variables are presented as absolute values and percentages. Continuous, normally distributed data are presented as means and standard deviations (SD). Between-group differences were determined by χ^2^ statistics or Fischer's exact tests, as appropriate, and unpaired two-sample *t* tests. Time intervals are presented as medians and interquartile ranges (IQR), and between-group differences were analyzed using the Mann-Whitney *U* test.

A *p* value <0.05 was used as level of significance. All statistical analyses were performed using SPSS 18.0.

## Results

### Patient cohort

A total of 74 patients (38 females and 36 males) underwent decompressive craniectomy. Their mean age was 49 years (range 6–78 years). Craniectomy was performed due to MCA infarction in 47 patients (63.5 %), aSAH in 17 (23 %), ICH or ASDH in seven (9.5 %), and reactive brain edema following tumor surgery in three (4.0 %). The craniectomy was unilateral in 73 patients and bifrontal in one. Fifty out of 74 patients (68.4 %) survived and had their bone flap replaced at a later point of time. Three patients were foreign tourists who had their bone flap replaced 4 to 26 days after decompressive craniectomy (before repatriation to their home country). None of them developed SSI during the hospital stay in Oslo. All three were lost to follow-up.

Thus, 47 patients, 20 females and 27 males with a median age of 47.8 years (range 6–74 years) were eligible for analysis and included in the study. Among these, decompressive craniectomy was performed for the following reasons: MCA infarction in 31 patient (66.0 %), aSAH in 11 (23.4 %), ICH or ASDH in four (8.5 %), and reactive edema after tumor surgery in one (2.1 %). Clinical data of the 47 patients are presented in Table 1. They were followed for a median of 41 months (range 2–155 months).

**Table 1:** Background characteristics of 47 patients who underwent delayed cranioplasty

	All *n* = 47	SSI *n* = 6	Non-SSI *n* = 41	*P*
Age, mean (SD)	47.8 (13.1)	48.2 (13.4)	47.7 (13.3)	0.94
Male sex	27 (57.4)	4 (66.7)	23 (56.1)	1.00
Smoking	12/39 (30.8)	2/5 (40.0)	10/34 (29.4)	0.63
Comorbidity				
Diabetes	5 (10.6)	1 (16.7)	4 (9.8)	0.51
Cancer^a^	5 (10.6)	0 (0)	5 (12.2)	1.00
COPD^b^	3 (6.4)	0 (0)	3 (7.3)	1.00
Cardiovascular disease	14 (29.8)	4 (66.7)	10 (24.4)	0.06
Cause of craniectomy				
MCA infarction	31 (66.0)	2 (33.3)	29 (70.7)	0.16
SAH	11 (23.4)	3 (50.0)	8 (19.5)	0.13
Tumor cerebri	1 (2.1)	1 (16.7)	0 (0)	0.13
ICH/ASDH	4 (8.5)	0 (0)	4 (9.8)	1.00
Right-sided	28 (59.6)	2 (33.3)	26 (63.4)	0.20
In-hospital infection after craniectomy^c^	40 (85.1)	5 (83.3)	35 (85.4)	1.00
Time from craniectomy to cranioplasty in days, median (IQR)	97 (82–120)	94 (74–164)	101 (83–121)	0.70
Operation time in min, median (IQR)	115 (95–159)	148 (110–204)	114 (90–142)	0.06
Prophylactic antibiotics	36 (76.6)	4 (66.7)	32 (78.0)	0.61
Post-operative subcutaneous drainage	16 (34.0)	3 (50.0)	13 (31.7)	0.40

Values are n and (%), unless indicated otherwise

^a^Known prior to craniectomy and considered stable disease: two patients with breast cancer, one with ovarian cancer, one with cervical cancer, and one with testicular cancer

^b^Chronic obstructive pulmonary disease

^c^Pneumonia, urinary infection, sepsis, and infection without known focus requiring postoperative antibiotic treatment

### Primary cranioplasty

The median time from decompressive craniectomy to primary cranioplasty was 97 days (range 38-568 days). The median duration of the surgical procedure was 115 min (range 40–230 min).

Two patients (4.3 %) underwent surgical removal of a postoperative epidural hematoma within 6 h after the cranioplasty. Their bone flaps were replaced immediately after evacuation of the hematoma. None of these two patients had a subcutaneous drainage placed in connection with the primary procedure.

### SSI, bone resorption, and bone flap removal

Six patients (12.8 %) developed SSI following replacement of the autologous cryopreserved bone. The bone flaps were removed 5, 17, 39, 119, 164, and 729 days, respectively, after the primary cranioplasty. The pathogens found and assumed to be responsible for the infections were: E. coli after 5 days, Propionibacterium acnes after 39 and 119 days, Methicillin-resistant Staphylococcus aureus after 164 days, and S. aureus after 17 and 729 days. Two of the six patients that developed SSI (P. acnes after 39 days and S. aureus after 17 days) had for unknown reasons not received prophylactic antibiotic treatment prior to replacement of their cryopreserved bone. However, there was no statistical significant difference in the rate of SSI among those who received prophylactic antibiotics and those who did not (*p* = 0.61) (Table 1).

All six patients that developed SSI were treated with antibiotics following antimicrobial susceptibility testing for 4 to 8 weeks after removal of the implanted bone.

Two patients, a 6-year-old boy and a 61-year-old woman, had their bone flaps removed due to bone resorption 1,645 and 1,000 days, respectively, after cranioplasty.

Seven of the eight patients who had their bone flaps removed later underwent a secondary cranioplasty using a patient-specific prosthesis made of porous, biocompatible polyethylene. The eighth patient died a few weeks before planned secondary cranioplasty. None of the patients developed SSI after the secondary cranioplasty.

### Patient groups and SSI resulting in bone flap removal

The background characteristics for all patients (*n* = 47) and the groups with (*n* = 6) or without SSI (*n* = 41) are presented in Table 1. There was no statistical significant difference between the two groups in any of the variables. There was, however, a trend towards more cardiovascular disease (SSI in 4/6 [67 %] versus non-SSI in 10/41 [24 %]; *p* = 0.06), and longer surgical procedure in the SSI group (median; SSI 148 min [IQR, 110–240 min] versus non-SSI 114 min [IQR, 90–142 min]; *p* = 0.06).

## Discussion

In the present study, eight out of 47 patients who underwent decompressive craniectomy and primary cranioplasty with autologous cryopreserved bone had their replaced bone flap removed again later due to SSI (six patients) and bone resorption (two patients). In the SSI group, there was a trend toward longer surgical procedure and more cardiovascular disease. None of the patients developed SSI after secondary cranioplasty using patient-specific cranial prosthesis.

### Preservation of bone flaps

Bone flaps removed during decompressive craniectomy are often preserved in a deep freezer, but the practice differs with regard to the freezing temperature used, ranging from −16 °C to −84 °C [2, 25, 26, 34, 37, 42]. Iwama et al. evaluated the complication rates at different freezing temperatures (−84 °C versus −35 °C). Among their 47 patients, one developed SSI and one experienced bone flap resorption, both occurring in the group with a storage temperature of −35 °C [26].

### Cranioplasty

Although various alloplastic materials such as stainless steel mesh-acrylic [15], titanium [27, 46], methyl methachrylate [4, 5], and customized prefabricated prostheses [9, 11, 13, 16, 47] are available for cranioplasty, most surgeons prefer to use the patient’s own bone whenever possible. A review of the most common mode of skull defect reconstruction in 25 major neurosurgical centers in Australia showed that 96 % preferred the use of cryopreserved autologous bone [3]. The use of autologous bone has the advantages of an ideal geometrical fit. Also, bone flaps kept frozen for up to 19 months have been shown to have the capacity for revitalization through a process of revascularization, resorption, and accretion [42]. Oh et al. demonstrated that osteoblast-like cells can survive in frozen bone [39], which concurs with clinical, radiologic, and radionuclide studies indicating the same [22].

### SSI after cranioplasty with cryopreserved bone

The use of cryopreserved autologous bone in the reconstruction of the cranial defect after craniectomy entails a risk for developing SSI. The infection rate varies between studies, from 0 to 25.9 % with an average of 7.7 % (Table 2) [2, 7, 19, 24–26, 32, 34–37, 42, 53].

**Table 2:** Reported SSI in cranioplasty with autologous bone

Author	N	Implant	Preservation	SSI
Moreira-Gonzales et al. [35]	312	Autologous bone	N/A	22 (7.1 %)
Matsuno et al. [34]	54	Autologous bone	Cryopreservation −20 °C	14 (25.9 %)
Iwama et al. [26]	37	Autologous bone	Cryopreservation −35 °C	1 (2.7 %)
	12	Autologous bone	Cryopreservation −84 °C	0 (0 %)
Inamasu et al. [25]	31	Autologous bone	Cryopreservation −70 °C	5 (16.1 %)
	39	Autologous bone	Subcutaneous pocket	2 (5.1 %)
Huang et al. [24]	151	Autologous bone	Cryopreservation −75 °C	5 (3.3 %)
Lu et al. [32]	16	Autologous bone	Cryopreservation −80 °C	0 (0 %)
Cheng et al. [7]	52	Autologous bone	Cryopreservation −?°C	7 (13.5 %)
Grossmann et al. [19]	12	Autologous bone	Cryopreservation −80 °C	0 (0 %)
Prolo et al. [42]	53	Autologous bone	Cryopreservation −20 °C	2 (3.7 %)
Asano et al. [2]	110	Autologous bone	Cryopreservation −40 °C	5 (4.5 %)
Nagayama et al. [37]	208	Autologous bone	Cryopreservation −16 °C	8 (3.88 %)
Zingale et al. [53]	21	Autologous bone	Cryopreservation −18 °C	0 (0)

*N*/*A* indicates not available

At some institutions, the removed bone flaps are routinely examined for bacterial contamination prior to storage. Chiang et al. found that the frequency of SSI did not increase after reimplanting bone flaps contaminated with microorganisms such as P. acnes, coagulase-negative Staphylococci, and S. aureus [8]. However, the risk of SSI in the reimplantation of bone flaps with positive bacterial cultures prior to preservation has not been determined. The same pathogens mentioned above were involved in five out of six cases of SSI in our patients. We do not know if they were contaminated before cryopreservation, since bacteriological examination of the bone flap prior to preservation has not been implemented as routine in our department.

We did not find any statistically significant relationship between the time interval from decompressive craniectomy to primary cranioplasty and the risk of SSI. This is in accordance with several previous studies [17, 41, 50], but in contrast with others, who have shown benefits from delaying the cranioplasty procedure [20, 43, 48]. However, we found a non-significant trend toward prolonged operation time being associated with an increased risk of SSI. This corresponds well with the findings in two recently published studies by Kim H et al. and Lee et al. [28, 30], but contrasts with the results of Tokoro et al [48]. According to Manson et al., the risk of SSI increases from about 5 % in routine primary cranioplasty to 14 % during a second cranioplasty [33]. None of our six patients developed SSI following secondary cranioplasty using patient-specific cranial prosthesis.

Prophylactic antibiotics were administered less often in the SSI group (67 %) compared to the non-SSI group (78 %) in our study. The difference was, however, not statistically significant. Prophylactic antibiotics are not standard treatment in our department and are therefore dependent on the surgeon’s preference. This is probably the reason why not all patients received prophylactic antibiotics.

In the present study, there was no association between the etiology of craniectomy and SSI following primary cranioplasty. Walcott et al. found, however, that stroke patients had a higher risk of SSI after cranioplasty than trauma patients [50], but no trauma patients were included in our study. Moreover, no statistically significant association was found between SSI and patient age, sex, prophylactic antibiotics or postoperative subcutaneous drainage. These findings correspond well with the results of Tokoro et al [48].

### Bone resorption after cranioplasty

Several authors have reported on the risk factors for developing bone resorption following cranioplasty with autologous bone [1, 2, 21, 38, 40, 42]. Hancock et al. found that 10 out of 12 sterilized, reimplanted bone flaps resorbed in children younger than 13 years [21]. Also, others have reported an increased risk of bone flap resorption in very young children [40, 42].

In our study, bone flap resorption occurred in a 6-year-old boy and in a 61-year-old woman 1,645 and 1,000 days, respectively, after cranioplasty. The follow-up period for all patients was median 41 months (range 2–155 months). One might suspect that a longer follow-up period in the present study could have revealed bone resorption in more patients. However, Schuss et al. found in their study that 60 % of bone flap resorption occurred within 1 year of follow-up and that no resorption was observed among patients followed for more than 5 years [45]. On the other hand, in a study by Grant et al. on patients younger than 19 years, bone resorption occurred in 20 out of 40 patients. Cranioplasty was performed with autologous bone following decompressive craniectomy and bone resorption occurred a mean 13.3 months (range 2–76 months) after primary cranioplasty [18].

Autoclaving the frozen bone before reimplantation in order to reduce the risk of SSI [1, 2, 21, 38, 40], along with shunt procedures [2] both seem to increase the risk of bone flap resorption.

### Limitations of our study

Our study is a retrospective analysis and may suffer from anticipated deficiencies related to the loss of patient information. Various neurosurgeons with possibly differing operative techniques and assessments of indication for surgery, as well as the heterogeneous patient population may have influenced the study results. In addition, the small sample size precludes firm conclusions about the risk factors related to SSI.

## Conclusion

SSI was the most common complication of the reimplantation of autologous cryopreserved bone after decompressive craniectomy seen in this study. Prolonged procedural time and cardiovascular comorbidity seemed to increase the risk of SSI. Because of the limited power of the study, it is, however, impossible to draw reliable conclusions, and further larger trials are required.
